# Examining Tympanostomy Tube Risk by Breastfeeding Duration: Is Six Months Enough?

**DOI:** 10.7759/cureus.101076

**Published:** 2026-01-08

**Authors:** Raj Patel, Germaine Harvey, Rebecca Maddrell

**Affiliations:** 1 Otolaryngology-Head and Neck Surgery, Loyola University Chicago Stritch School of Medicine, Maywood, USA; 2 Family Medicine, Loyola University Medical Center, Maywood, USA

**Keywords:** breastfeeding duration, early-life immune protection, passive immunity, recurrent acute otitis media, tympanostomy tube

## Abstract

Background: Breastfeeding provides passive immunoglobulin A (IgA) immunity to newborns, supporting proper sinonasal, pulmonary, and mucosal protection. Current recommendations advise breastfeeding for at least six months before introducing complementary foods, with continuation encouraged for up to two years. Infants who are not breastfed are at increased risk for sinopulmonary infections, including acute otitis media. Recurrent or chronic acute otitis media can lead to tympanostomy tube placement. While this highlights the protective role of breastfeeding, there is limited data on whether extending breastfeeding beyond six months offers additional protection against the need for tympanostomy tube insertion.

Objective: This study aimed to evaluate whether longer durations of breastfeeding provide additional protection against tympanostomy tube placement in pediatric patients.

Methods: A retrospective chart review was conducted to evaluate pediatric patients who underwent tympanostomy tube placement using Current Procedural Terminology (CPT) codes 69436 and 69433 and a control group matched by age and gender. Feeding type was classified as formula-fed or breastfed, with breastfeeding duration recorded to the best of available documentation. Children who received both breast milk and formula were considered breastfed. Chi-squared tests and risk ratios were used to analyze the association between breastfeeding duration and tympanostomy tube placement.

Results: Among 500 pediatric patients, breastfeeding duration was significantly associated with tympanostomy tube insertion (χ²=9.36; p=0.025). Children breastfed for less than six months or not at all had a tube insertion rate of 20.8% (reference). Those breastfed for six months had a rate of 11.6% (RR: 0.72; p=0.025). Children breastfed for more than six months had a rate of 8.4% (RR: 0.79; p=0.08). Those breastfed for more than 12 months had a rate of 9% (RR: 0.95; p=0.08). Pairwise comparisons between children breastfed for six months and those breastfed for more than six months (χ²=0.24; p=0.63) or more than 12 months (χ²=2.99; p=0.084) were not statistically significant.

Conclusion: Breastfeeding for up to six months is associated with a lower risk of tympanostomy tube insertion compared to children breastfed for shorter than six months or not breastfed at all. Continuing breastfeeding beyond six months and 12 months does not appear to provide additional protective benefit.

## Introduction

Infancy is marked by rapid immune development alongside heightened vulnerability to infection. Endogenous immunoglobulin (Ig) production is limited early in life, and maternal immunoglobulin G (IgG) transferred transplacentally provides systemic protection for only the first 6-12 months. Infants' own IgG levels do not reach approximately two-thirds of adult concentrations until around 12 months of age [1]. Mucosal immunity is even more delayed, as secretory immunoglobulin A (sIgA) remains low during the first months of life and reaches more mature levels only after the first year [2]. This period of immunological immaturity leaves infants reliant on exogenous sources of mucosal defense. Human breast milk uniquely supplies high concentrations of sIgA, lactoferrin, lysozyme, human milk oligosaccharides (HMOs), cytokines, and antimicrobial peptides (AMPs), which together compensate for the infant's underdeveloped mucosal immune system [3].

The protective effects of breast milk on infant respiratory and gastrointestinal health are well established. sIgA in human milk neutralizes viral and bacterial pathogens at mucosal surfaces, limits adherence to respiratory epithelium, and shapes the developing airway microbiome which are mechanisms that are directly relevant to acute otitis media (AOM) [2,4]. By reducing respiratory infections and attenuating nasopharyngeal and Eustachian tube inflammation, breastfeeding lowers the risk of middle ear effusion and subsequent AOM [5].

AOM is among the most common pediatric infections, with most children experiencing at least one episode by three years of age and recurrent disease posing a persistent challenge for families and clinicians [6]. Tympanostomy tube placement is the most frequently performed pediatric surgery in the United States, with over 700,000 procedures annually and cumulative incidence estimates indicating that 6-9% of children undergo tube insertion in early childhood [7,8]. Although tympanostomy tubes can reduce persistent effusion and improve hearing, they are associated with certain risks including otorrhea, repeat tube placement, tympanic membrane changes, and anesthesia exposure. These considerations underscore the importance of preventative strategies at the clinical and public health levels [9].

Breastfeeding is a modifiable factor influencing infant infection risk, and both the American Academy of Pediatrics and the World Health Organization recommend exclusive breastfeeding for approximately six months, followed by continued breastfeeding with complementary foods [10,11]. Observational studies consistently demonstrate that breastfeeding, particularly exclusive breastfeeding, reduces AOM risk. Meta-analyses report a 23-50% reduction in AOM incidence among infants who are ever breastfed, with the strongest protective effect observed in those exclusively breastfed for the first 4-6 months of life [12,13]. Exclusive breastfeeding for longer than six months has also been associated with lower rates of recurrent AOM [14].

Despite evidence that breastfeeding reduces AOM risk, it remains unclear whether breastfeeding beyond six months provides additional protection against tympanostomy tube placement, a more clinically meaningful outcome. Prior studies often use broad breastfeeding categories or lack precise duration definitions, limiting the assessment of incremental benefit and leaving the relationship between extended breastfeeding and tube placement insufficiently defined [15,16]. This study addresses this gap by evaluating whether breastfeeding beyond six months is associated with reduced tympanostomy tube placement, with the goal of clarifying the role of breastfeeding and the rate of tympanostomy tube insertion associated with it. 

This retrospective chart review differs from prior studies by stratifying infants into four distinct breastfeeding duration groups (<6 months or not breastfed, six months, >6 to <12 months, and ≥12 months), allowing for a more detailed assessment of duration-dependent effects. Unlike most previous work, it examines tympanostomy tube placement as a clinically meaningful outcome rather than just AOM incidence or recurrence. Additionally, this study incorporates socioeconomic status (SES) using zip code-level adjusted gross income to explore disparities in breastfeeding practices and their impact on tube insertion rates. These features provide a more nuanced understanding of how breastfeeding duration and SES together influence the risk of tympanostomy tube placement.

## Materials and methods

A retrospective chart review was conducted of pediatric patients treated within Loyola University Medical Center, Maywood, Illinois, between 2015 and 2025. Patients who underwent tympanostomy tube placement were identified using Current Procedural Terminology (CPT) codes 69436 and 69433. A control cohort was selected from pediatric patients within the same health system and study period who had no documented history of tympanostomy tube placement. Controls were required to have longitudinal clinical follow-up within the health system to ensure sufficient opportunity for tympanostomy tube placement had it been clinically indicated, thereby reducing the misclassification of patients with incomplete care records. All medical records were de-identified prior to analysis.

An initial cohort of 759 patients meeting the inclusion criteria was identified. Patients were matched by age and sex to minimize confounding related to developmental stage and sex-based differences in otitis media risk. From this matched cohort, 250 male and 250 female patients were randomly selected to comprise the final study population for detailed manual chart review.

Breastfeeding exposure was categorized into four predefined and mutually exclusive duration groups: breastfed for less than six months or not breastfed, breastfed for six months, breastfed for more than six months but less than 12 months, and breastfed for 12 months or longer. Feeding type was classified as breastfed or formula-fed based on documentation in well-child visits and clinical notes. Patients who received both breast milk and formula were classified as breastfed. Breastfeeding duration was recorded using the most complete documentation available in the medical record. When breastfeeding was documented at or beyond a specific well-child visit, duration was assigned accordingly. If breastfeeding was not documented beyond the six-month visit, duration was categorized as six months. This approach was used to ensure consistent classification across patients, recognizing that incomplete documentation may result in some exposure misclassification.

SES was estimated using zip code-level adjusted gross income data as a proxy measure. Patient zip codes were linked to corresponding adjusted gross income values derived from publicly available data sources. Patients were stratified into three socioeconomic categories representing lower income, lower middle income, and upper middle income groups based on income distribution within the study population. Median adjusted gross income was calculated for each category. Zip code-level income was used as an area-based estimate of SES, acknowledging that this method does not capture individual-level variation in household income, education, or access to resources.

Statistical analyses were performed using chi-squared tests of independence to compare breastfeeding duration groups and to assess associations between SES and tympanostomy tube placement. Relative risks and corresponding confidence intervals were calculated to estimate the strength of association between breastfeeding duration and tympanostomy tube insertion. Analyses were conducted as unadjusted comparisons due to the inconsistent documentation of key clinical and environmental confounders, which limited the feasibility of multivariable regression modeling. Statistical significance was defined as a two-sided p-value of less than 0.05.

## Results

A total of 759 pediatric patients were included in the analysis. After matching by age and sex, the final study population included 250 males and 250 females (Table 1). Gender distribution was evenly balanced post-matching, with 50% male and 50% female patients, ensuring comparability across breastfeeding groups.

**Table 1:** Gender distribution between pre- and post-matching

Gender	Pre-match	Chi square (χ²)	P-value	OR	95% CI	Post-match	P-value
Male	438	χ²=6.92	p<0.01	1.36	1.09-1.71	250	Not applicable
Female	321			0.74	0.58-0.92	250	

The overall chi-squared test demonstrated a statistically significant association between breastfeeding duration and tympanostomy tube insertion (χ²=9.36; p=0.025) (Table 2). The tube insertion rate was highest among children breastfed for less than six months or not breastfed (20.8%), which served as the reference group. In comparison, tympanostomy tube insertion occurred in 11.6% of children breastfed for six months (RR=0.72; 95% CI: 0.57-0.91), 8.4% of those breastfed for longer than six months (RR=0.79; 95% CI: 0.61-1.03), and 9% of those breastfed for longer than 12 months (RR=0.95; 95% CI: 0.74-1.20) (Table 2). Pairwise comparisons revealed no significant difference between children breastfed for six months and those breastfed for longer than six months (χ²=0.24; p=0.024) or between children breastfed for longer than 12 months and those breastfed for six months (χ²=2.99; p=0.06) (Table 3).

**Table 2:** Breastfeeding duration and tympanostomy tube insertion

Groups	No tympanostomy tube insertion	Tympanostomy tube insertion	Total	Tube insertion rate	Relative risk vs. formula	95% CI
Breastfed for <6 months + not breastfed	84	104	188	20.8%	1.0	Not applicable
Breastfed for 6 months	88	58	146	11.6%	0.72	CI: 0.57-0.91
Breastfed for >6 months	54	42	96	8.4%	0.79	CI: 0.61-1.03
Breastfed for >12 months	41	45	86	9%	0.95	CI: 0.74-1.20
Total	250	250	500	Not applicable		

**Table 3:** P-value differences among breastfeeding duration

Group	Chi square (χ²)	P-value	OR	95% CI
Overall	χ²=9.36	p=0.025	Not applicable	Not applicable
Breastfed for 6 months vs. breastfed for >6 months	χ²=0.24	p=0.024	0.85	0.50-1.43
Breastfed for 6 months vs. breastfed for >12 months	χ²=2.99	p=0.06	0.60	0.35-1.03

SES analysis demonstrated that children from higher SES backgrounds were more likely to be breastfed for longer durations. Among children breastfed for six months, 72 were from higher-class, 54 from middle-class, and 20 from lower-class households. In contrast, the majority of children breastfed for less than six months or not breastfed were from lower-class households (117), with fewer from middle-class households (42) and higher-class households (29) (Table 4). The chi-squared analysis confirmed a significant association between SES and breastfeeding duration (p<0.01).

**Table 4:** SES distribution SES: socioeconomic status

SES class	Breastfed for <6 months + not breastfed	Breastfed for 6 months	Breastfed for >12 months	Breastfed for >12 months	Chi square (χ²)	P-value
Higher	29	72	54	48	χ²=163.3	p<0.01
Middle	42	54	37	29		
Lower	117	20	5	9		
Total	188	146	96	86		

## Discussion

Breastfeeding provides essential nutrients and immunologic protection during an infant's early months of life. Among these protective factors, sIgA plays a central role in defending the sinopulmonary mucosa against infection. Maternal antibodies transferred transplacentally and through breast milk gradually decline and reach a nadir at approximately six months of age [1,2]. During this period, infants begin producing endogenous antibodies, though immunologic maturity is not reached until later in infancy, with IgG levels approaching two-thirds of adult concentrations by 12 months of age [17]. This window of immunologic vulnerability overlaps with the peak incidence of AOM, which occurs most frequently between six and 18 months of age and may progress to recurrent disease requiring tympanostomy tube placement [17]. Understanding the association between breastfeeding duration and tympanostomy tube insertion is therefore clinically relevant.

In this retrospective cohort, infants who were breastfed for less than six months or not breastfed had the highest incidence of tympanostomy tube insertion. Breastfeeding for six months was associated with a significantly lower incidence of tube placement and a reduced relative risk compared with the reference group. Children breastfed for longer than six months demonstrated similarly lower rates of tube insertion, although these associations did not reach statistical significance in pairwise comparisons. While the overall association between breastfeeding duration and tympanostomy tube insertion was statistically significant, extending breastfeeding beyond six months was not associated with a statistically significant additional reduction in risk. These findings suggest that breastfeeding for at least six months is associated with a meaningful reduction in tympanostomy tube placement, while longer durations may maintain a similar level of protection. Importantly, these findings reflect associations rather than causal relationships and should be interpreted accordingly. The observed protective association with breastfeeding for at least six months aligns with the American Academy of Pediatrics recommendations emphasizing the immunologic benefits of breastfeeding during early infancy [10,11].

Analysis of breastfeeding duration across socioeconomic strata demonstrated a strong association between higher SES and longer breastfeeding duration. This relationship highlights SES as a potential confounding factor, as socioeconomic and healthcare access variables may influence both breastfeeding practices and the likelihood of tympanostomy tube placement. Parents in lower socioeconomic groups may face substantial barriers to sustained breastfeeding, including longer work hours, limited access to paid parental leave, fewer workplace accommodations for pumping, and reduced access to lactation support services [18]. Educational, cultural, and psychosocial factors may further influence breastfeeding behaviors, as well as healthcare utilization and referral patterns. Because SES is also associated with access to pediatric care and specialist referral, it may contribute to observed differences in tympanostomy tube placement independent of otitis media severity. Although SES was descriptively analyzed in this study, it was not incorporated into adjusted statistical models, and residual confounding related to socioeconomic and healthcare access factors remains an important consideration.

Several limitations of this study warrant discussion. First, the retrospective observational design inherently limits causal inference and increases susceptibility to residual confounding. Second, the accurate determination of breastfeeding duration through chart review was challenging due to incomplete or inconsistent documentation. Breastfeeding may have continued beyond what was recorded in the medical record, resulting in potential misclassification. To ensure internal consistency, breastfeeding duration was categorized based on documentation at well-child visits; however, some degree of nondifferential misclassification is likely. Third, SES was estimated using adjusted gross income at the zip code level, which may not accurately reflect individual household circumstances and can obscure variability in education, occupation, healthcare access, and social support.

Additionally, several key confounding variables known to influence the risk of otitis media and tympanostomy tube placement, including daycare attendance, household smoking exposure, prematurity, and medical comorbidities, were inconsistently documented in the electronic health record. As a result, these variables could not be reliably extracted or incorporated into adjusted analyses. This limitation contributed to the reliance on unadjusted chi-squared analyses and limited the feasibility of multivariable regression modeling. While chi-squared testing was appropriate for identifying group-level associations in this exploratory analysis, the absence of multivariable modeling limits the ability to assess whether breastfeeding duration is independently associated with tympanostomy tube placement after accounting for correlated socioeconomic and clinical factors. Consequently, the observed associations may reflect the influence of unmeasured or incompletely measured confounders rather than an isolated effect of breastfeeding duration.

Despite these limitations, this study provides clinically relevant evidence supporting an association between breastfeeding for at least six months and a lower incidence of tympanostomy tube placement. Future prospective studies with standardized documentation of breastfeeding practices, comprehensive capture of socioeconomic and environmental variables, and multivariable modeling will be important to better delineate the independent contributions of breastfeeding duration, SES, and healthcare access to otitis media severity and surgical intervention risk.

## Conclusions

Breastfeeding for at least six months is associated with a reduced risk of tympanostomy tube insertion compared to children who were either not breastfed or breastfed for less than six months. The relative risk reduction appears strongest at six months of breastfeeding, with a protective effect that diminishes with longer durations, as the risk in those breastfed for longer than 12 months approaches that of the reference group. Additionally, a shorter duration of breastfeeding is associated with lower SES classes. 
